# Risk factors for Kienböck’s disease and need for surgical intervention: a nationwide register study from Finland

**DOI:** 10.1177/17531934251387061

**Published:** 2025-10-30

**Authors:** Krista Wernér, Turkka Anttila, Timo Viljakka, Jorma Ryhänen, Sina Hulkkonen

**Affiliations:** 1 Musculoskeletal and Plastic Surgery, Department of Hand Surgery, University of Helsinki and Helsinki University Hospital, Helsinki, Finland; 2 Tampere University Hospital, Tampere, Finland

**Keywords:** Kienböck disease, risk factor, epidemiology, bone avascular necrosis, surgical intervention, immunosuppression, operation, systemic disorders

## Abstract

**Introduction:**

This study was designed to investigate the association between previously known risk factors for other avascular necrosis disorders of bone and Kienböck’s disease and the need for surgical intervention.

**Methods:**

Details of patients with Kienböck’s disease and possible predisposing factors were obtained from multiple nationwide Finnish health registers for the period 1996–2022.

**Results:**

Men were more often affected and had a higher risk for operations, with an incidence rate ratio 1.24 (95% CI: 1.15 to 1.33) and a risk ratio of 1.16, respectively. Many previously known risk factors for other avascular necrosis were found to be associated with Kienböck’s disease, such as alcohol abuse (odds ratio (OR) 1.67), rheumatic diseases (OR 1.81), coagulopathic state (OR 1.59) and history of hand and wrist injuries (OR 3.03). Operated patients had a higher prevalence of type 1 diabetes (OR 2.43) and coagulopathic risk factors (OR 1.40). Patients requiring total wrist arthrodesis had a higher prevalence of immunosuppression (OR 1.77), cardiovascular risk factors (OR 2.31), alcohol abuse (OR 2.13), rheumatoid arthritis (OR 3.74), pancreatitis (OR 3.03) and hypertension (OR 1.94).

**Conclusions:**

Many systemic disorders were found to be associated with Kienböck’s disease. Wrist and hand injuries might also be predisposing factors. Factors such as immunosuppression, alcohol abuse and cardiovascular risk factors may increase the risk of developing a more severe form of this disease, requiring surgery.

**Level of evidence:**

III

## Introduction

Kienböck’s disease (KD) is a rare condition where the lunate is affected by avascular necrosis (AVN). It can affect people of all ages and is seen predominantly in men (Wernér et al., 2025; White et al., 2016). Kienböck’s disease is considered to be multifactorial, and certain biomechanical and anatomical factors generate the ‘at-risk’ lunate described by Bain et al. (2016). These factors include vascular variations (Gelberman et al., 1980; Panagis et al., 1983), lunate morphology (Rhee et al., 2015) and radial inclination (Thienpont et al., 2004). The connection of KD to negative ulnar variance has been investigated widely, but insufficient evidence exists to support causality (Chung et al., 2001; Stahl et al., 2013). ‘At-risk’ lunate is believed to be more prone to developing KD owing to the repetitive microtrauma (Bain et al., 2016). On the other hand, Stahl et al. (2012) found inadequate evidence to support the causality of KD and hand–arm vibration in their systematic review. Kienböck’s disease has rarely been linked to lunate or perilunate dislocations (Takami et al., 1996) or primary fractures (Teisen and Hjarbaek, 1988), which also argues against a solely traumatic aetiology.

Few studies have investigated the association between KD and metabolic factors, which have been previously linked to AVN of other bones. For instance, coagulopathies, sickle cell anaemia, hyperlipidaemia, autoimmune disorders, use of corticosteroids and excessive alcohol intake have been associated with AVN of the femoral head (George and Lane, 2022; Lohiya et al., 2023; Shah et al., 2015). Some studies have reported an association between KD and these metabolic factors or systemic disorders, but most articles are case reports (Frerix et al., 2016; Kazmers et al., 2020; Taniguchi et al., 2002b). Camus and Van Overstraeten (2022) postulate that the biomechanical and anatomical factors could be predisposing factors to lunate fractures and collapse rather than the cause of the necrosis itself. To conclude, the focus for future research should be to elucidate the role of these biological and metabolic factors in causing necrosis.

Previous research suggests that the natural evolution of the disease may vary (Keith et al., 2004; Kristensen et al., 1986; Stahl et al., 2014; Viljakka et al., 2016). Some patients might be somewhat asymptomatic, in contrast to others who undergo multiple operations because of persisting pain. Furthermore, incidental findings of asymptomatic, advanced-stage KD demonstrate that clinical findings do not always correlate with radiological ones (van Leeuwen et al., 2016; Taniguchi et al., 2002a). Very little is currently known about factors explaining variation in disease severity, except for the patient’s age (Lichtman et al., 2016).

Data from the Care Register for Health Care controlled by the Finnish Institute for Health and Welfare has previously been analysed to report on the incidence rate of KD in Finland and to assess the surgical procedures that are used for KD (Wernér et al., 2025). The current study used this dataset in addition to accessory data from the Care Register for Health Care, the Social Insurance Institution of Finland and the Digital and Population Data Services Agency and aimed to investigate the association between previously known risk factors for other AVNs and KD and the need for surgical intervention.

## Methods

Our data were collected between 1 January 1996 and 31 December 2022 from several registers: (1) the Care Register for Health Care, controlled by the Finnish Institute for Health and Welfare (THL); (2) the Social Insurance Institution of Finland (Kela); and (3) the Digital and Population Data Services Agency (DVV). This dataset included some data that were used for our previous study (Wernér et al., 2025) but was complemented by a substantial amount of additional information. Finnish registers are comprehensive and reliable and enable high-quality epidemiologic studies (Sund, 2012). The Finnish Social and Health Data Permit Authority (Findata) processed and combined the data from different registers, pseudonymized the data, and approved the publication of the results (THL/2563/14.02.00/2022).

Patients with KD were identified with the International Classification of Diseases (10th revision, ICD-10) codes M93.1 (Kienböck’s disease of adults) and M92.2 (Juvenile osteochondrosis, hand). Four controls for each patient with the same sex and age ± 2 years were collected from the DVV register. For two patients, DVV was unable to obtain control patients either owing to an inactive personal security number or non-disclosure for personal safety reasons. Altogether, 1394 KD patients and 5576 controls were included in this study. Supplementary Table S1 shows codes for entitlement to reimbursement for medicine expenses collected from Kela’s register. Supplementary Tables S2 and S3 present selected permanent diagnoses and wrist or hand trauma diagnoses, respectively. All authors reached consensus on included diagnoses based on previously known risk factors for AVN. These diagnoses and all operation data were collected from THL’s registers, which cover all specialized health care in Finland.

We analysed the association of different diagnoses with four terminal events: KD diagnosis; operations; multiple operations; and total wrist arthrodesis. Similar diagnoses were combined into subgroups based on consensus of the authors (Table 1). Continuous data (age) are presented as median and interquartile range (IQR), and normality was assessed with the Shapiro–Wilk test as well as visual inspection of histograms. The association of age at diagnosis with different terminal events was analysed with the Mann–Whitney *U*-test owing to non-normal distribution. Categorical variables (sex, diagnoses) are presented as frequencies and the association of sex with terminal events was evaluated using a Chi-squared test. Incidence rate ratio was used to assess the difference in risk for KD between the sexes with the exact Poisson test.

**Table 1.** Combination of diagnoses for logistic regression analyses.

Group of diagnoses	Included diagnoses
Blood coagulation	Alcohol abuse, smoking, hypercoagulopathies, pancreatitis, vasculitis, chronic kidney diseases, atherosclerosis, systemic sclerosis
Cardiovascular risk factors	Atherosclerosis, hypertension, hyperlipidaemia, diabetes, smoking
Coagulopathic state	Hypercoagulopathies, vasculitis, smoking, pancreatitis, diabetes, chronic kidney diseases, systemic sclerosis
Connective tissue diseases	Polymyalgia rheumatica, systemic sclerosis, vasculitis, dermatomyositis, systemic lupus erythematosus
Diseases with cortisone	Asthma, colitis ulcerosa/Crohn, transplantation, all rheumatic diseases, chronic kidney diseases, Cushing syndrome
Haematological malignancies	Leukaemias, lymphomas
Immunosuppression	All rheumatic diseases, transplantation, colitis ulcerosa/Crohn, chronic kidney diseases, haematological malignancies, alcohol abuse, asthma, amyloidosis
Rheumatic diseases	Rheumatoid arthritis, juvenile idiopathic arthritis, polymyalgia rheumatica, ankylosing spondylitis, psoriasis, vasculitis, dermatomyositis, systemic lupus erythematosus, systemic sclerosis, gout
*Injuries*	
Fractures	Scaphoid fracture, fracture of other carpal bone, fracture of other/unclassified area of wrist or hand, distal radius fracture, distal antebrachial fracture, ulnar shaft fracture
Contusions	Contusion of other part of wrist and hand, wrist sprain or distension, sprain or distension of other/unclassified part of wrist or hand, other wrist or hand injury, unclassified wrist and/or hand injury
Ligament injuries	Wrist dislocation, wrist and/or hand traumatic ligament rupture

Univariate logistic regression was employed to assess the association between different diagnoses and KD using a ratio of four controls to one affected individual. All diagnoses were included to evaluate the difference in lifetime prevalence. Sensitivity analyses were carried out to confirm that adding sex and age to the analyses would not affect the results. The correlation between different variables and KD is represented as an odds ratio (OR). Age and sex were added to multivariable logistic regression analyses as confounding factors to assess the OR for different diagnosis combinations and other terminal events. Possible multicollinearity was evaluated with variance inflation factor, and a value >5 was considered a significant multicollinearity. Patients with multiple operations were compared with patients with only one operation, and total wrist arthrodesis patients were compared with patients with other operations. A *p*-value < 0.05 was considered significant in all analyses.

## Results

Compared with women, men had 24% more KD cases per person-years (incidence rate ratio = 1.24, 95% CI: 1.15 to 1.33, *p* < 0.001) and 16% higher risk (*p* = 0.02) for an operation when diagnosed with KD. Numbers for these calculations was provided by our previous study (Wernér et al., 2025). Sex had no association with the risk of having multiple operations (*p* = 0.61) or total wrist arthrodesis (*p* = 0.84). The median age at KD diagnosis was significantly lower (*p* = 0.01) for patients who ended up with operations than for patients with no operations, 40 (IQR: 28 to 51) and 43 (IQR: 27 to 57), respectively. In contrast, the median age at KD diagnosis was higher (*p* = 0.03) for patients requiring total arthrodesis than for those with other operations, 43 (IQR: 35 to 49.5) and 39 (IQR: 27 to 51), respectively. The age at diagnosis had no association with the risk for multiple operations (*p* = 0.99). Table 2 presents ORs with 95% CIs for different diagnoses, with each column for different terminal events. No disease was significantly associated with the risk for multiple operations, and thus, this was omitted from the table.

**Table 2.** Univariate/multivariable logistic regression model results for analysing different predisposing factors for different terminal events. Only results with significant odds ratios *p* < 0.05 are shown. Data are expressed as odds ratio (95% confidence interval). Patients/controls are expressed as frequencies.

Group of diagnoses	Kienböck’s disease	Patients/controls (1394/5576)	Operations (608/786)	Total arthrodesis (71/537)
Alcohol abuse	1.67 (1.34 to 2.07)	124/307		2.13 (1.05 to 4.09)
Alcohol abuse and pancreatitis	1.86 (1.51 to 2.27)	147/332	1.50 (1.06 to 2.13)	2.08 (1.07 to 3.86)
Ankylosing spondylitis		12/24		
Asthma	1.65 (1.36 to 2.00)	157/397		
Atherosclerosis	2.18 (1.50 to 3.11)	46/86		
Biological medication	1.82 (1.32 to 2.48)	58/130		
Blood coagulation	1.98 (1.66 to 2.35)	214/467		
Cardiovascular risk factors	1.50 (1.32 to 1.70)	464/1392		2.31 (1.36 to 3.94)
Cerebral palsy	6.42 (2.14 to 21.28)	8/5		
Chronic kidney diseases	2.08 (1.06 to 3.92)	14/27		
Coagulopathic state	1.59 (1.36 to 1.86)	258/696	1.40 (1.06 to 1.86)	
Colitis ulcerosa/Crohn		29/103		
Connective tissue diseases	2.14 (1.31 to 3.43)	26/49		
Diabetes	1.43 (1.21 to 1.70)	208/607		
Diabetes type 1	1.64 (1.11 to 2.37)	39/96	2.43 (1.27 to 4.85)	
Diseases with cortisone	1.72 (1.48 to 2.00)	295/750		
Haematological malignancies		16/50		
Hyperlipidaemia	1.68 (1.38 to 2.03)	160/399		
Hypertension	1.41 (1.22 to 1.62)	332/1012		1.94 (1.10 to 3.37)
Immunosuppression	1.71 (1.51 to 1.94)	527/1457		1.77 (1.06 to 2.97)
Juvenile idiopathic arthritis	3.48 (1.63 to 7.35)	13/15		
Pancreatitis	2.83 (1.75 to 4.50)	30/43		3.03 (0.94 to 8.49)
Rheumatic diseases	1.81 (1.45 to 2.24)	128/295		
Rheumatoid arthritis	2.03 (1.35 to 2.98)	38/76		3.74 (1.10 to 11.41)
Systemic sclerosis	5.35 (1.86 to 16.28)	8/6		
				
*Injuries*				
Fractures	2.33 (1.87 to 2.89)	137/249		
Contusions	3.74 (2.74 to 5.10)	79/88		
Ligament injuries	8.80 (5.35 to 14.91)	47/22		
Dislocation	12.03 (2.77 to 82.17)	<10		
All injuries	3.03 (2.53 to 3.61)	234/348		

Almost all studied diagnoses were found to be associated with KD (Table 2). The highest OR was with cerebral palsy and systemic sclerosis but with wide confidence intervals owing to few cases. A narrower confidence interval was found with hypertension, immunosuppression, coagulopathic state, and diabetes. These diagnoses, together with cardiovascular risk factors, alcohol abuse and rheumatoid arthritis were also associated with more severe disease, requiring operations. Hand and wrist injuries were associated with KD but not with operations.

## Discussion

We found that many previously known risk factors for bone AVN seem to be associated with KD, such as hyperlipidaemia, several autoimmune diseases, coagulation disorders and history of injury to the affected body area/site. Alcohol abuse/pancreatitis, diabetes type 1, coagulopathic state, male sex and lower age at KD diagnosis were associated with need for surgery. Immunosuppression, cardiovascular risk factors, alcohol abuse, pancreatitis, hypertension, rheumatoid arthritis and higher age at KD diagnosis were more common in patients requiring total arthrodesis. We will next discuss the previous literature regarding risk factors in relation to our findings.

In accordance with our results, previous studies have demonstrated the relationship between AVN of the femoral head and several systemic disorders (Bhayana et al., 2024; Boechat et al., 2001; Giertz et al., 2024; Lohiya et al., 2023; Shah et al., 2015; Zhao et al., 2015). An association with KD has previously been reported only for history of smoking, diabetes, glucocorticoid use and alcohol use (Kazmers et al., 2020). There are also case reports suggesting that cerebral palsy, gouty arthritis, systemic lupus erythematosus, systemic sclerosis and juvenile idiopathic arthritis could be associated with KD, but no conclusion can be drawn without more data (Chouhan et al., 2020; Desy et al., 2011; Frerix et al., 2016; Gallien et al., 2010; Kazmers et al., 2020; Mok et al., 1997). To our knowledge, no earlier study has examined the association of cardiovascular risk factors with AVN. For diabetes, the evidence is limited (Konarski et al., 2022). In our study, almost all of the included diagnoses seemed to be associated with KD. This raises the question of whether KD patients really have more long-term diseases than the general population or whether this finding reflects that a patient complaining of wrist pain during a visit to specialized health care is more likely to be diagnosed with KD.

Previous studies have discovered an association between autoimmune diseases and AVN but have not extrapolated this finding to other conditions with an immunocompromised state (Lohiya et al., 2023). In our study, immunosuppression was more common in KD patients and also in patients who required total arthrodesis. This finding suggests that immunosuppression is also an important factor regarding disease severity. An intriguing question arises: could infections be a contributing factor to the development of AVN? This hypothesis is bolstered by emerging evidence showing that COVID-19 increases the incidence of osteonecrosis, an effect not entirely explained by glucocorticoid use (Murugesan et al., 2024; Sakellariou et al., 2024; Za et al., 2025). Moreover, some other infections have also been linked to AVN (Huang et al., 2024; Kanno et al., 2024). These observations warrant further investigation.

Several studies have linked AVN of the femoral head to fractures of the femoral neck and hip dislocations (Shah et al., 2015). In contrast, evidence for trauma increasing the risk of KD is scarce (Desmarais and Soong, 2013; Singh and Reese, 2024). Based on our study, hand and wrist injuries were much more common in KD patients than in controls (OR = 3.03), and ligament injuries seemed to be especially dominant (OR = 8.80). Our findings align with those of previous studies concerning AVN of the femur. Hypothetically, from a circulation perspective, it makes sense to think that ligament injuries could lead to either direct vascular injuries or oedema caused by trauma could cause vascular impairment and venous congestion of the lunate. Nevertheless, it is important to point out that oedema seen after wrist injuries could easily be transient type such as bone marrow oedema syndrome, previously described as a differential diagnosis for the AVN of the femur (Cui et al., 2021). These could present as false positives in our data and could lead to overestimation of the association.

Certain anatomical and biomechanical risk factors have been suggested to be predisposing factors for collapse of the lunate in KD, but biological risk factors and systemic diseases might be the true cause of development of AVN (Camus and Van Overstraeten, 2022). Our findings also support this idea since multiple systemic diseases were found to associate with KD. One interesting possibility is that osteonecrosis of bone could be caused by necroptosis, a form of programmed cell death activated by multiple factors such as infection, injury or inflammation (Khoury et al., 2020). This process involves damage-associated molecular patterns, previously shown to be present in osteonecrosis of the femoral head (Deng et al., 2022). The potential role of necroptosis in the development of AVN and KD should be investigated further.

Very little is known about explanatory factors for the severity of KD or any other AVN (Rhee et al., 2015; van Leeuwen et al., 2017; Xu et al., 2013). Based on our findings, coagulopathic and cardiovascular diseases, immunosuppression, alcohol abuse, rheumatoid arthritis and pancreatitis were more common in severe disease, requiring operations. Patients requiring total arthrodesis also had higher age at diagnosis. As a potential pathogenesis, the role of endothelial dysfunction and damage-associated molecular patterns in osteonecrosis of the femoral head is explained by Shao et al. (2024). Dysfunction can be caused by reactive oxygen species, which can be provoked by, for instance, glucocorticoids, alcohol abuse, hypercholesterolaemia, diabetes and hypertension (Shao et al., 2024). Interestingly, many of these factors seem to contribute to more severe KD.

Our study’s strength is a comprehensive, nationwide register that has proven to be reliable in epidemiological studies (Sund, 2012). In Finland, health care is available for everyone regardless of financial or insurance status, which reduces possible bias. We looked for associations with a wide range of diagnoses and extended the search with reimbursement codes for medical expenses from Kela. Nevertheless, some diagnoses have probably been missed, particularly since certain diseases are primarily diagnosed in primary healthcare, which was not covered in this register. Also, factors such as smoking and alcohol abuse are probably not completely covered in the register. However, the diagnoses obtained were filtered similarly for patients and controls, compensating for this possible bias. Furthermore, although we utilized a nationwide register, some diagnoses are so rare and surgically treated KD patients are such a small group that it is impossible to draw any conclusions from them. The major limitation of our study is that the true causal relationship between risk factors and different outcome events cannot be evaluated owing to the study’s retrospective case–control design. Avascular necrosis in general and especially KD are so rare that prospective cohort studies are arduous to conduct. However, our study provides valuable information from a nationwide register about plausible associations with this rare disease and offers insights into potential factors affecting the severity of KD.

Men appear to be at a higher risk for KD and need for surgical intervention. Many systemic disorders previously associated with other AVN seem also to contribute to ‘at-risk’ lunate for KD. Contrary to previous findings, wrist and hand injuries may also be predisposing factors. Our findings suggest that factors such as immunosuppression, alcohol abuse and cardiovascular risk factors might increase the risk of developing a more severe form of this disease, requiring operations. Understanding more of the mechanisms by which these systemic factors may cause and aggravate necrosis could bring new possibilities for early diagnosis, handling of risk factors, treatment and maybe eventually, even prevention of KD.

## Supplemental Material

sj-docx-1-jhs-10.1177_17531934251387061 – Supplemental material for Risk factors for Kienböck’s disease and need for surgical intervention: a nationwide register study from Finland Supplemental material, sj-docx-1-jhs-10.1177_17531934251387061 for Risk factors for Kienböck’s disease and need for surgical intervention: a nationwide register study from Finland by Krista Wernér, Turkka Anttila, Timo Viljakka, Jorma Ryhänen and Sina Hulkkonen in Journal of Hand Surgery (European Volume)

sj-docx-2-jhs-10.1177_17531934251387061 – Supplemental material for Risk factors for Kienböck’s disease and need for surgical intervention: a nationwide register study from Finland Supplemental material, sj-docx-2-jhs-10.1177_17531934251387061 for Risk factors for Kienböck’s disease and need for surgical intervention: a nationwide register study from Finland by Krista Wernér, Turkka Anttila, Timo Viljakka, Jorma Ryhänen and Sina Hulkkonen in Journal of Hand Surgery (European Volume)

sj-docx-3-jhs-10.1177_17531934251387061 – Supplemental material for Risk factors for Kienböck’s disease and need for surgical intervention: a nationwide register study from Finland Supplemental material, sj-docx-3-jhs-10.1177_17531934251387061 for Risk factors for Kienböck’s disease and need for surgical intervention: a nationwide register study from Finland by Krista Wernér, Turkka Anttila, Timo Viljakka, Jorma Ryhänen and Sina Hulkkonen in Journal of Hand Surgery (European Volume)
