# Supplementary material for: Risk factors for Kienböck’s disease and need for surgical intervention: a nationwide register study from Finland
Source: J Hand Surg Eur Vol. 2025 Oct 30;51(4):415–21. doi: 10.1177/17531934251387061 (PMC12967377; doi:10.1177/17531934251387061)
Supplement: sj-docx-1-jhs-10.1177_17531934251387061 – Supplemental material for Risk factors for Kienböck’s disease and need for surgical intervention: a nationwide register study from Finland [file sj-docx-1-jhs-10.1177_17531934251387061.docx]

**Table S1.** Codes for entitlement to reimbursement for medicine expenses.

| Medicine | Disease | Code |
| --- | --- | --- |
| Dasatinib | Leukaemia | 150 |
| Nilotinib | Leukaemia | 152 |
| Bosutinib | Leukaemia | 170 |
| Imatinib | Leukaemia | 189 |
|  | Leukaemia and other haematological malignancies | 117 |
| Benralizumab, mepolizumab | Asthma | 251, 297 |
|  | Chronic asthma and similar chronic obstructive lung diseases | 203 |
| Abatacept, adalimumab, anakinra, etanercept, golimumab, ixekizumab, infliximab, sarilumab, secukinumab, certolizumab pegol, tocilizumab | Rheumatism, arthritis | 281 |
| Baricitinib, upadacitinib | Rheumatoid arthritis | 293, 298, 3029 |
| Abatacept, adalimumab, anakinra, etanercept, golimumab, ixekizumab, infliximab, sarilumab, secukinumab, certolizumab pegol, tocilizumab, ustekinumab | Rheumatic diseases | 303 |
| Tofacitinib | Arthritis | 291 |
| Tofacitinib | Rheumatoid arthritis/colitis ulcerosa | 3005 |
|  | Connective tissue diseases, rheumatic arthritis, or similar conditions | 202 |
| Adalimumab, golimumab, infliximab, ustekinumab  vedolizumab | Crohn disease/colitis ulcerosa | 326, 3042 |
|  | Colitis ulcerosa and Crohn disease | 208 |
| Dulaglutide, exenatide, lixisenatide, liraglutide, semaglutide | Diabetes type 2 | 285, 346 |
|  | Diabetes, insulin | 103 |
| Febuxostat | Gout | 288 |
|  | Gout | 212 |
| Febuxostat | Chronic hyperuricaemia | 349 |
|  | Organ or tissue transplant | 127 |
|  | Chronic hypertension | 205 |
| Nalmefene, naltrexone | Alcohol/opioid addiction | 309 |
| Eliglustat, imiglucerase, velaglucerase alfa | Gaucher disease | 151, 333 |
| Colesevelam, alirokumab, evolocumab | Familial hypercholesterolaemia | 292, 345, 388 |
|  | Severe hereditary lipid metabolism disorders (familial hypercholesterolaemia, type III dyslipoproteinaemia) | 211 |
| Metyrapone, ketoconazole | Cushing disease | 365, 3021 |
