# Supplementary material for: Risk factors for Kienböck’s disease and need for surgical intervention: a nationwide register study from Finland
Source: J Hand Surg Eur Vol. 2025 Oct 30;51(4):415–21. doi: 10.1177/17531934251387061 (PMC12967377; doi:10.1177/17531934251387061)
Supplement: sj-docx-2-jhs-10.1177_17531934251387061 – Supplemental material for Risk factors for Kienböck’s disease and need for surgical intervention: a nationwide register study from Finland [file sj-docx-2-jhs-10.1177_17531934251387061.docx]

**Table S2.** Permanent diagnoses and their codes according to the International Classification of Diseases (10^th^ revision, ICD-10; 9^th^ revision, ICD-9; 8^th^ revision, ICD-8).

| Diagnosis | ICD-10 | ICD-9 | ICD-8 |  |
| --- | --- | --- | --- | --- |
| Alcohol abuse | F10.xx | 303 | 303 |  |
| Amyloidosis | E85.xx | 277.3x | 276 |  |
| Ankylosing spondylitis | | M45 | 720.0 | 726, 727 |
| Asthma | J45.0, J45.1, J45.8, J45.9 | 493.x | 493 |  |
| Atherosclerosis | I70.xx | 440 | 440 |  |
| Cerebral palsy | G80.xx | 343.x |  |  |
| Chronic kidney diseases | | N03#, N08*, N11.x, N16* | 585.x | 582 |
| Crohn disease and Colitis ulcerosa | K50.xx, K51.xx | 555, 556, 558.9 | 563 |  |
| Cushing disease | E24.xx | 255.0 | 255 |  |
| Dermatomyositis | M33.9 | 710.3 | 716 |  |
| Diabetes | E10.xx, E11.xx, E13.xx, E14.xx | 250.x0, 250.x1, 250.x2, 250.x3 | 250 |  |
| Gaucher disease | E75.2 | 272.7 | 272 |  |
| Gout | M10.xx | 274 | 274 |  |
| Human immunodeficiency virus | B20.xx, B21.xx, B22.xx, B23.xx | 042, 043, 044 |  |  |
| Hypercoagulopathies | D68.8 | 289.81 | 286 |  |
| Hyperlipidaemia | E78.xx | 272.0, 272.1, 272.2, 272.3, 272.4 | 272 |  |
| Hypertension | I10 | 401 | 401 |  |
| Hyperuricaemia | E79.0 |  |  |  |
| Juvenile idiopathic arthritis | M08.x | 714.3x |  |  |
| Leukaemia | C94.xx, C95.xx, C91.xx, C92.xx | 204.x, 205.x, 206.x, 207.x, 208.x | 204-207 |  |
| Lymphoma | C81.xx, C82.xx, C83.xx, C85.xx | 201.x, 202.x | 201, 202 |  |
| Organ transplantation | Z94.0, Z94.1, Z94.2, Z94.3, Z94.4 | V42.0, V42.1, V42.6, V42.7 | 446 |  |
| Pancreatitis | K85.xx | 577.0, 577.1 | 577 |  |
| Polymyalgia rheumatica | M35.3 | 725 | 716, 717 |  |
| Psoriasis | L40.x, M07.x*, M09.0* | 696.0, 696.1 | 696 |  |
| Rheumatoid arthritis | M05.xx, M06.xx | 714 | 712 |  |
| Sickle cell anaemia | D57.x | 282.6x | 282 |  |
| Smoking | F17.xx, Z72.0 | 289.81 |  |  |
| Systemic sclerosis | M34.xx | 710.1 |  |  |
| Thalassaemia | D56.xx | 282.4x | 282 |  |
| Vasculitis | M30.xx, M31.xx | 446.x |  |  |
