# Supplementary material for: Risk factors for Kienböck’s disease and need for surgical intervention: a nationwide register study from Finland
Source: J Hand Surg Eur Vol. 2025 Oct 30;51(4):415–21. doi: 10.1177/17531934251387061 (PMC12967377; doi:10.1177/17531934251387061)
Supplement: sj-docx-3-jhs-10.1177_17531934251387061 – Supplemental material for Risk factors for Kienböck’s disease and need for surgical intervention: a nationwide register study from Finland [file sj-docx-3-jhs-10.1177_17531934251387061.docx]

**Table S3.** Wrist and hand trauma diagnoses and their codes according to the International Classification of Diseases (10^th^ revision, ICD-10; 9^th^ revision, ICD-9; 8^th^ revision, ICD-8),

| Diagnosis | ICD-10 | ICD-9 | ICD-8 |
| --- | --- | --- | --- |
| Contusion of other part of wrist and hand | S60.2 |  |  |
| Scaphoid fracture | S62.0 | 814 | N814 |
| Fracture of other carpal bone | S62.1 | 814 | N814 |
| Fracture of other/unclassified area of wrist or hand | S62.8 | 818 | N818 |
| Wrist dislocation | S63.0 | 833 | N833 |
| Wrist and/or hand traumatic ligament rupture | S63.3 |  |  |
| Wrist sprain or distension | S63.5 | 842.01, 842.02, 842.09 | N842 |
| Sprain or distension of other/unclassified part of wrist or hand | S63.7 | 842.00 |  |
| Crush injury of other/unclassified part of wrist or hand | S67.8 | 927.2 |  |
| Multiple wrist and/or hand injuries | S69.7 | 923.8 |  |
| Other wrist or hand injury | S69.8 | 923.2 | N925 |
| Unclassified wrist and/or hand injury | S69.9 | 923.9 |  |
| Distal radius fracture | S52.5 | 813.4, 813.5 | N813 |
| Distal antebrachium fracture | S52.6 | 813.4, 813.5 | N813 |
| Ulnar shaft fracture | S52.2 | 813.22, 813.32 | N813 |
